# Hypertension and its associated factors in Hosanna town, Southern Ethiopia: community based cross-sectional study

**DOI:** 10.1186/s13104-018-3435-1

**Published:** 2018-05-16

**Authors:** Likawunt Samuel Asfaw, Samuel Yohannes Ayanto, Fiseha Laemengo Gurmamo

**Affiliations:** 1 Department of Nursing, Hosanna College of Health Sciences, Post Box 159, Hosanna, Ethiopia; 2 Department of Midwifery, Hosanna College of Health Sciences, Hosanna, Ethiopia; 3 Hosanna College of Health Sciences, Hosanna, Ethiopia

**Keywords:** Cardio-vascular disorders, Hypertension, Non-communicable diseases, Obesity

## Abstract

**Objectives:**

This study was conducted to determine the prevalence of hypertension and its associated factors among residents of Hosanna town in Hadiya Zone.

**Results:**

The overall prevalence of hypertension was 30% among the study participants. Out of the study participants who were identified as being hypertensive, only 24.6% knew their hypertensive status. The odds of being hypertensive is significantly higher among males when compared to females (adjusted odds ratio (AOR) 1.9, confidence interval (CI) 1.14–3.23) and married participants as compared to their unmarried counterparts (AOR 4.1; CI 1.10–16.18). High prevalence and increased risks for hypertension were noted among the study participants in the study area. The experiences of aerobic physical activities were reported only in 22.9% of the study participants. These evidences may suggest the need for urgent interventions.

## Introduction

Hypertension is persistent elevation of BP above this normal range [1] and is classified into different groups based on causes and degree of severity [2–5].

Hypertension has become major public health problem of human being globally. It is estimated to cause 1 billion deaths, which is about 12.8% of all deaths worldwide [6, 7]. In Africa 46% of its adult population had hypertension, which is the highest for regions in the world [6, 8]. Similarly, the figure for sub-Saharan Africa was 47.5% [9, 10].

Ethiopia shares similar profile like most sub-Saharan African countries. Findings of World Health Organization on prevalence of hypertension showed that 35.2% of the community in Ethiopia has high likelihood of being hypertensive [6]. To a large extent hypertension is associated with environmental, rapid urbanization and life style changes [11, 12]. There are controversial opinions on the association between hypertension and gender. In prevalence study in rural Bareilly there was no significant difference between males and females [13, 14]. However, in most global and Ethiopian studies hypertension is more prevalent among males than females [14–16]. Obesity, tobacco smoking and harmful alcohol use are significantly associated with hypertension [17–25]. Majority of previous studies done in Ethiopia were based on hospital records and reported contradicting opinions. Therefore, the aim of this study was to assess the prevalence and associated factors of hypertension in a community sample.

## Main text

### Methods

#### Study design and setting

The study was conducted in Hosanna town, the capital of Hadiya Zone, located at a distance of 232 km southwest of Addis Ababa, the capital of Ethiopia. There were 16,707 Households in the town. Community based cross-sectional study was carried out among residents of the town, in May 2014 [26].

#### Sample size and sampling technique

The desired sample size for our study was estimated by taking prevalence of hypertension (35.2%) from previous study [6], 95% confidence level, 5% margin of error and design effect of 1.5. Consequently, the final sample size was determined to be 525 participants. The sample size was calculated using the formula;

$${\text{n}} = \frac{{{\text{Z}}\frac{\alpha }{2}^{2 } {\text{P}}(1 - {\text{P}})}}{{{\text{d}}^{2} }}$$

$${\text{n}} = \frac{{\left( {1.96 } \right)^{2 } 0.35(1 - 0.35)}}{{(0.05)^{2} }} = 350 (1.5) = 525$$

The final sample size was proportionally allocated to sub-administrative units of the town. Sampling frame was created for each sub-unit and randomly generated numbers were used to select the households. Simple random sampling technique was used to select the households from each unit. From each of the selected households, one participant satisfying inclusion criteria was selected by lottery method.

#### Inclusion and exclusion criteria

Individuals below the age of 25 years, those above the age of 64 years, pregnant mothers and disabled people were excluded from the study. The primary reason for excluding pregnant women and individuals above the age of 64 is that they are most at risk for hypertension and their inclusion could preclude generalization. Contrarily, young people below the age of 25 years are at low risk for hypertension and disabled people were not eligible for exercise related inquiries relevant for our research which might affect the true finding in the population.

#### Data collection instrument and measurement

The WHO STEPS instrument and global physical activity questionnaire (GPAQ) were modified and used [27, 28]. The tool has three major parts: socio-demographic characteristics, behavioral profile and physical measurements. The modified instrument was translated into the local language, Amharic. Data were collected through interviewer administered and physical body measurement techniques using structured questionnaire.

Two days training was provided for data collectors and supervisors regarding research ethics, data collection procedures and contents of the instrument to increase the quality of our data. Supportive supervision was carried out by the supervisors on a daily basis during the data collection period. The completed questionnaire had been checked daily for its completeness and consistency.

The blood pressure was measured after the participant had rested for at least 5 min. Two measurements at 10 min interval were taken from right arm by a mercury sphygmomanometer. The mean value of the two measurements was recorded as a BP for each participant.

Height was measured using fixed height measuring board in upright position with participant’s heel, shoulder and buttock touching the vertical board behind. The measurement value was recorded to the nearest millimeter. Weight was measured using calibrated weight scale where participants being in light clothing and barefooted. Its reading was taken to the nearest 0.1 kg. Waist circumference measurement was taken at midpoint between lower measure margin of the last plain rib and top of iliac crest using non elastic tape meter. Each participant was told to take little deep, natural breath before taking the measurement. The measurement was taken at the end of normal expiration, when the lungs are at their residual capacity.

#### Data analysis techniques

The collected data were cleaned and entered to Epi-Data version 3.2, and exported to STATA version 12.0 for analysis. Descriptive statistics and multivariable logistic regression were used to analyze the data. Candidate variables with P value < 0.2 in Bivariable model were entered to multivariable model to adjust for predictors. The 95% CI for the corresponding Odds Ratio (OR) was used to assess the degree of associations at (P < 0.05) to declare significance.

#### Variables and definitions

The participant was regarded as hypertensive when an average SBP ≥ 140 mmHg, and/or DBP ≥ 90 mmHg was recorded and/or the participant is currently on antihypertensive medications.

The body mass index (BMI) was interpreted according to WHO classification as underweight (BMI < 18.5 kg/m^2^), normal (BMI 18.5–24.9 kg/m^2^), overweight (BMI 25.0–30.0 kg/m^2^) and obese (BMI > 30.0 kg/m^2^).

Men having waist circumference greater than 94 cm were identified as having increased risk for hypertension and metabolic complications whereas men having waist circumference greater than 102 cm were identified as having substantially increased risk for hypertension and metabolic complications.

Women having waist circumference greater than 80 cm were identified as having increased risk for hypertension and metabolic complications whereas women having waist circumference greater than 88 cm were identified as having substantially increased risk for hypertension and metabolic complications.

## Results

A total of 524 participants were involved in the study which gives response rate of 99.8%. The majority (52.9%) of study participants were males. The mean age of the study participants was 35.4 ± 7.7 SD years. Majority (38.5%) of participants were government employee. Nearly half (48.8%) of the study participants were College or University graduates. The average monthly income of the study participants was 72.31 ± 916.33 USD. The average number of individuals per household was nearly 6 (Table 1). One hundred twenty-two (23.3%) participants reported their experience of alcohol consumption on daily basis and 77 (14.7%) participants were smokers.

**Table 1:** Socio-demographic characteristics of study participants in Hosanna town 2014

Characteristics	Number	Percent
Sex (n = 524)		
Male	277	52.9
Female	247	47.1
Age (n = 524)		
25–34	102	19.5
35–44	273	52.1
45–54	110	21.0
55–64	39	7.4
Marital status (n = 524)		
Single	195	37.1
Married	192	36.6
Divorced	106	20.2
Widowed	31	5.9
Education (n = 524)		
No formal education	24	4.6
Primary education	130	24.8
Secondary education	115	21.9
College/university	255	48.7
Income (n = 524)		
< 54 USD	132	25.2
55–91 USD	233	44.5
92–109 USD	30	5.7
> 109 USD	129	24.6

### Physical measurements

**Fig. 1:**
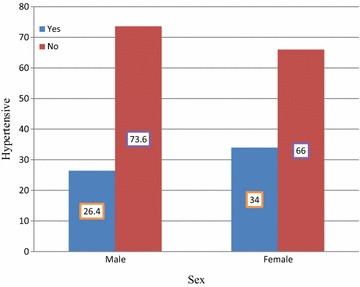
Distribution of hypertension by sex of participants

The mean systolic and diastolic BP reading for the study participants were 118.37 ± 13.42 (SD) mmHg and 74.24 ± 11.18 (SD) mmHg respectively. The prevalence of hypertension among the study participants was 30% (CI 26.0–33.8%) out of which only 39 (24.6%) knew their hypertensive status (Fig. 1).

The mean BMI of the study participants was 23.79 ± 2.60 kg/m^2^ (SD). Fifty-four (10.3%) participants were overweight and 28 (5.3%) were obese.

The mean waist circumference of men was 86.9 ± 4.9 cm (SD). The vast majority (84.1%) of men had waist circumference measurement of ≤ 94 cm whereas 36 (12.9%) of them had > 94 cm. Few, 3.0%, of male participants had > 102 cm waist circumference measurement. The findings indicate that 12.9% men had increased risk for hypertension and metabolic complications.

The mean waist circumference for women was 83.6 ± 8.35 SD cm. One hundred four (42.1%) women had waist circumference measurement of < 80 cm. Nearly one out of three (29.1%); women have waist circumference measurement of > 80 cm. On the other hand, 71 (28.7%) of women had waist circumference measurement of > 88 cm. This finding represents that more than half of women had higher Waist circumference measurements. Twenty-nine percent of women had increased risk for hypertension and metabolic complications and 28.7% of women had substantially increased risk for hypertension and metabolic complications. Overall, 37.2% (15.8% men and 61.1% women) participants were identified as having increased risk for hypertension and metabolic complications.

### Physical activity

Fifty-seven (10.9%) and 108 (20.6%) of the study participants undertake vigorous and moderate-intensity physical activities respectively. Only 22.9% of the participants reported the experience of aerobic physical activities. The average estimated time spent without movement among the study participants was 10.25 ± 3.1 (SD) hours.

### Factors associated with hypertension

The presence or absence of significant association between independent and outcome variables was determined. Accordingly, sex, marital status and aerobic physical activities were significantly associated with hypertension. The odds of hypertension was 1.92 times higher among men as compared to females (AOR 1.92; CI 1.14–3.23). The likelihood of hypertension was significantly higher among married participants when compared to unmarried ones (AOR 4.1; CI 1.0–16.18). Participants who did not undertake aerobic physical activities had three times more likely to develop hypertension as compared to participants who did (AOR 3.0; CI 1.1–6.5) (Table 2).

**Table 2:** Bivariate and multivariate logistic regression analysis of factors associated with hypertension in Hosanna (n = 524), Ethiopia, 2014

Characteristics	Hypertension		Bivariate OR (95% CI)	Multivariate OR (95% CI)	P-value
	Yes	No			
Sex					
Male	73	204	1.4 (0.99,2.06)	1.92 (1.14,3.23)	0.014^†^
Female	84	163	1	1	0.00
Marital status					
Single^a^	37	158	1	1	0.00
Married	64	128	2.03 (0.8,4.6)	4.1 (1.0,16.18)	0.04^†^
Divorced	46	60	0.95 (0.4,2.14)	1.7 (0.3,7.3)	0.46
Widowed	10	21	0.62 (0.2,1.4)	1.2 (0.18,7.8)	0.80
Aerobic physical activity					
Yes	137	267	1	1	0.00
No	20	100	2.5 (1.5,4.32)	3.0 (1.4,6.5)	0.004^†^
Age group					
25–34	19	83	1	1	0.00
35–44	75	198	0.6 (0.3,1.06)	0.4 (0.23,0.81)	0.01^†^
45–54	54	56	0.2 (0.12,0.4)	0.1 (0.04,0.25)	< 0.001^†^
55–64	9	30	0.7 (0.3,1.8)	0.4 (0.15,1.15)	0.09

1 = Reference

^a^Not ever married

^†^P-value < 0.05

## Discussion

The overall prevalence of hypertension in this study was 30.0%. This is comparable with report from Addis Ababa (31.5%) [25]. But higher than reported for Jimma town (13.2%) [29], Gondar town (28.3%) [12] and Sidama Zone (9.9%) [30]. However, the prevalence of hypertension in our study was lower than the national average (35.2%) [6] and Africa sub-regional prevalence (47.5%) [9, 10]. The national average was higher because it used health facility reports in its estimation, which might have not represented the true magnitude in the general public. This difference could be explained by differences in life style factors including diet, exercise and the use of different substances, etc. Among hypertensive participants, those participants who are aware of their hypertensive status were 24.6%, which matches with other study findings [28, 29]. This might indicate low awareness and screening practices for hypertension in the community.

The prevalence of hypertension was significantly higher among men participants than females. In contrast, previous study reports showed that the variation in occurrence of hypertension between the two sexes was not statistically significant [12]. Conversely, the higher rate of hypertension among men in our study was congruent with previous study reports [7, 9, 14, 16]. More likely, this difference could be explained as large number of men engage in risky behaviors such as excess alcohol consumption, smoking tobacco products, and ka’hat chewing that predispose to hypertension when compared to females. However, the controversy between reports on association between sex and hypertension warrant further study.

The prevalence of hypertension was found to be higher among higher age groups in previous studies [25, 29–31]. In our study age was obtained subjectively which might not be participants’ exact age due to absence of birth certificates in majority of the cases. Although the risk of hypertension increased with advancing age because of biological reasons, substance use in younger age groups balanced the prevalence of hypertension across all age groups. These facts could also, more likely, explain the importance of hypertension at any age.

In our study, marital status is significantly associated with hypertension and married participants were more likely to develop hypertension when compared to their unmarried counterparts. Community-based study in Jazan region of Saudi Arabia also reported the presence of association between marital status and hypertension [9]. This could be explained in such a way that married couples are vulnerable to and face disputes from different life dimensions. These stressful life conditions they may face could increase the risk of hypertension among them.

## Conclusion

High prevalence of hypertension was noted among the study participants. Only few participants were aware of their hypertensive status. The community is at increased risk for hypertension and metabolic complications. Women had substantially increased risk for hypertension when compared to males. Sex, marital status and limited exercise were significantly associated with hypertension. Increased prevalence of hypertension and its associated factors imply the need for urgent intervention by designing strategies to increase public awareness on risks, preventive measures and screening behaviors.

## Limitations

This study is cross-sectional; therefore, we cannot ascribe causality to any of the associated factors. Moreover, prevalence may not be representative as some severe cases may die soon after they develop the disease.

## Abbreviations

BMI: body mass index
CI: confidence interval
CVD: cardiovascular disorders
DALY: disability adjusted life year
EDHS: ethiopia demographic and health survey
GPAQ: global physical activity questionnaire
HBP: high blood pressure
HSDP: health sector development
MMHG: millimeter mercury
NCD: non-communicable diseases
PA: physical activity
SBP: systolic blood pressure
SSA: sub-Saharan Africa
WC: waist circumference
WHO: World Health Organization
